# Electronic cigarette use and risk of chronic kidney disease: a dose-response analysis with propensity score matching in a nationally representative cohort

**DOI:** 10.1186/s12889-025-24026-y

**Published:** 2025-08-27

**Authors:** Song Li, Ruoxuan Liu, Jianhua Zhang, Weidong Pei, Junqing Hou

**Affiliations:** 1 Department of Urology, Kaifeng155 Hospital, RongTong Medical Healthcare Group Co.Ltd, Kaifeng, 475000 China; 2 Department of Surgery, Huaihe Hospital of Henan University, Kaifeng, 475000 China; 3 Discipline Construction Department, RongTong Medical Healthcare Group Co.Ltd, Chengdu, 610000 China

**Keywords:** E-cigarette, Chronic kidney disease, Dose-response, Causal inference, Public health

## Abstract

**Background:**

The long-term renal effects of electronic cigarette (e-cigarette) use remain poorly understood, despite its global rise as a nicotine delivery system. This study investigates the association between e-cigarette use and chronic kidney disease (CKD), with emphasis on dose-response relationships, subgroup heterogeneity, and adjustment for cigarette smoking.

**Methods:**

We analyzed data from 872 adults in NHANES (2017–2020). E-cigarette use was categorized by self-reported frequency (non-users, 1–2 days/week, ≥ 3 days/week). CKD was defined as urinary albumin-to-creatinine ratio ≥ 30 mg/g. Weighted multivariable logistic regression models adjusted for age, gender, race, BMI, diabetes, hypertension, and current cigarette smoking. Propensity score matching (PSM, 1:1) balanced baseline covariates, including current smoking. Dose-response trends were assessed via restricted cubic splines (RCS).

**Results:**

Among 188 e-cigarette users and 684 non-users, users were significantly younger and had higher serum cotinine. In fully adjusted models (Model 3), e-cigarette use was associated with a 2.50-fold higher odds of CKD (95% CI: 1.80–3.48, *P* < 0.001). A significant dose-response relationship existed: adjusted ORs for CKD were 1.80 (95% CI: 1.20–2.70) for 1–2 days/week and 2.60 (95% CI: 1.70–4.00) for ≥ 3 days/week use (P-trend = 0.002). This association was significantly stronger in non-diabetics (OR = 2.40, 95% CI: 1.65–3.50) vs. diabetics (P-interaction = 0.032) and in current smokers (OR = 2.35, 95% CI: 1.55–3.56) vs. non-smokers (P-interaction = 0.021). Sensitivity analyses confirmed robustness: PSM yielded OR = 1.89 (95% CI: 1.18–3.01, *P* = 0.009); excluding dual users yielded OR = 1.98 (95% CI: 1.28–3.06, *P* = 0.003); and cotinine-based exposure yielded OR = 2.25 (95% CI: 1.43–3.54, *P* = 0.001).

**Conclusion:**

E-cigarette use is independently associated with CKD in a dose-dependent manner, particularly among non-diabetic individuals. Notably, NHANES data limitations include lack of past e-cigarette use or smoking history records, which may introduce residual confounding. These findings highlight vaping as a modifiable risk factor for kidney disease, urging targeted public health interventions.

## Introduction

The rapid global proliferation of electronic cigarette (e-cigarette) use, particularly among adolescents and young adults, has raised significant public health concerns regarding its long-term health effects. Emerging evidence suggests mixed effects of e-cigarette use on cardiopulmonary health, with some studies noting potential risks while others highlight harm reduction compared to traditional cigarettes [1–3]. However, their impact on renal health, particularly the risk of chronic kidney disease (CKD), remains poorly understood. CKD, a condition affecting over 10% of the global population, is a major contributor to cardiovascular morbidity and mortality, underscoring the urgent need to identify modifiable risk factors [4].

Nicotine and its metabolites, such as cotinine, are known to induce oxidative stress, endothelial dysfunction, and systemic inflammation—pathways implicated in the pathogenesis of CKD [5, 6]. While traditional cigarette smoking is an established risk factor for kidney damage [7], the role of e-cigarettes, which deliver nicotine without combustion byproducts, remains controversial. Recent animal studies suggest that e-cigarette aerosols exacerbate renal fibrosis and albuminuria [8], yet human epidemiological evidence is sparse and inconsistent. A critical gap exists in understanding whether e-cigarette use independently associates with CKD in a dose-dependent manner and whether this relationship varies across clinically relevant subgroups, such as individuals with diabetes or obesity. Diabetes and obesity are established CKD risk factors [4, 9], making subgroup analyses critical to understand effect modification. Diabetes may alter renal injury pathways via insulin resistance, while obesity drives inflammatory stress [10].

This study uses data from the National Health and Nutrition Examination Survey (NHANES), a nationally representative cohort, to address three key questions:

1. Is e-cigarette use independently associated with CKD after adjusting for demographic, metabolic, and clinical confounders?
2. Does a dose-response relationship exist between e-cigarette use frequency and CKD risk?
3. Are these associations modified by diabetes status, obesity, or current cigarette smoking?

By integrating advanced causal inference methods—including propensity score matching (PSM) and stratified analyses—this work aims to clarify the potential renal toxicity of e-cigarettes. Our findings have direct implications for public health policies and clinical guidelines aimed at mitigating CKD risk in vulnerable populations.

## Materials and methods

### Study population and data extraction

**Fig. 1:**
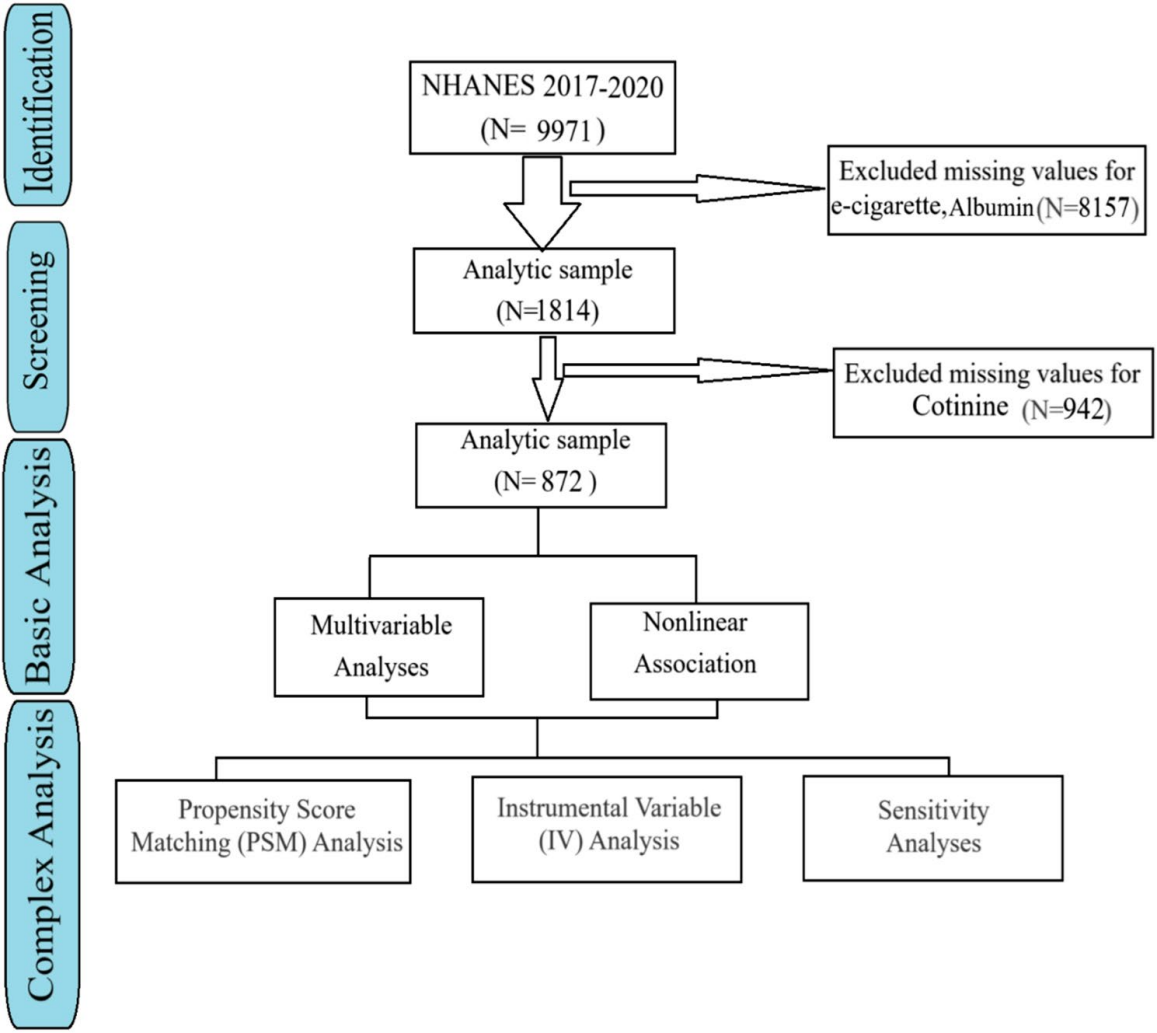
A flowchart of this study design

The study used data from the National Health and Nutrition Examination Survey (NHANES) spanning cycles 2017–2020, a nationally representative cross-sectional survey conducted by the Centers for Disease Control and Prevention (CDC). Participants aged ≥ 18 years with complete data on e-cigarette use, chronic kidney disease (CKD) status, current cigarette smoking status (yes/no), and covariates (age, gender, race, diabetes, BMI, serum cotinine, and systolic blood pressure) were included. CKD was defined as an estimated glomerular filtration rate (eGFR) < 60 mL/min/1.73 m² or urinary albumin-to-creatinine ratio (UACR) ≥ 30 mg/g. E-cigarette users were identified as individuals reporting ≥ 1 day of e-cigarette use in the past 30 days. Non-users were defined as those reporting no e-cigarette use in the past 30 days. BMI was calculated from measured height and weight per NHANES protocols. NHANES sampling weights (WTMEC2YR) were applied to account for complex survey design and ensure national representativeness. Ethical approval was obtained by NHANES, and all participants provided written informed consent(Fig. 1).

### Multivariable logistic regression

Weighted multivariable logistic regression models were constructed to assess the association between e-cigarette use and CKD, adjusting for potential confounders in a stepwise manner:

- Model 1 Adjusted for age (continuous), gender (male/female), and race/ethnicity (Mexican American, Other Hispanic, Non-Hispanic White, Non-Hispanic Black, Other Race).
- Model 2 Model 1 + body mass index (BMI, continuous, per 5 kg/m² increment).
- Model 3 Model 2 + diabetes, hypertension, and current cigarette smoking (yes/no).

Continuous variables were scaled to clinically meaningful units (e.g., age per decade). Variance inflation factors (VIF) confirmed no multicollinearity (VIF < 2.0). Analyses were performed using R v4.3.1 with the survey package to incorporate sampling weights. Statistical significance was set at *P* < 0.05 (two-tailed).

### Dose-Response relationship

To evaluate the dose-response relationship, e-cigarette use frequency was categorized as 0 days (non-users), 1–2 days/week, and ≥ 3 days/week based on self-reported usage. To address confounding from cigarette smoking, analyses were stratified into four groups: (1) Neither e-cigarettes nor cigarettes; (2) E-cigarettes only; (3) Cigarettes only; (4) Both e-cigarettes and cigarettes. Trend significance was assessed using the Cochran-Armitage test. Restricted cubic splines (RCS) with 3 knots (10th, 50th, 90th percentiles) were applied to model nonlinear associations. Linear trends were confirmed via likelihood ratio tests comparing linear and nonlinear spline models. Sensitivity analyses included: Alternative frequency categorization (0, 1–5, ≥ 6 days/week); Exclusion of dual users (concurrent traditional cigarette smokers); Substitution of self-reported frequency with serum cotinine levels (≥ 10 ng/mL as exposure threshold). Sensitivity analyses included a four-group classification based on current use of e-cigarettes and cigarettes to assess independent effects.

### Subgroup analysis

Subgroup analyses were conducted to evaluate potential effect modification by diabetes status and obesity. Participants were stratified into the following subgroups:

*Diabetes status*: Defined as self-reported physician-diagnosed diabetes, use of glucose-lowering medications.

*Obesity status*: Classified as BMI < 30 kg/m² (non-obese) or BMI ≥ 30 kg/m² (obese), consistent with WHO criteria.

*Current smoking status*: Current smokers (yes/no).

Interaction terms (e-cigarette use × subgroup variable) were incorporated into fully adjusted logistic regression models to test for heterogeneity. The significance of interactions was assessed using likelihood ratio tests comparing models with and without interaction terms. Adjusted odds ratios (ORs) and 95% confidence intervals (CIs) were reported for each subgroup.

### Sensitivity analysis

To ensure the robustness of the primary findings, comprehensive sensitivity analyses were conducted using weighted NHANES data with covariate adjustments consistent with the main models. First, serum cotinine (≥ 10 ng/mL) was used as exposure, excluding dual users (*n* = 156) to minimize confounding from traditional smoking. Second, the definition of chronic kidney disease (CKD) was modified to stricter diagnostic criteria (UACR ≥ 300 mg/g) to validate outcome consistency. Third, self-reported e-cigarette use was replaced with log-transformed serum cotinine levels as a continuous biomarker to objectively quantify nicotine exposure. Finally, propensity score matching (1:1) used age, gender, race, BMI, diabetes, systolic BP, and current cigarette smoking to balance covariates. Sensitivity analyses included a four-group classification based on current use of e-cigarettes and cigarettes to assess independent effects. All sensitivity analyses preserved the primary study’s statistical framework while systematically addressing potential confounding from smoking status, outcome definitions, exposure misclassification, and baseline characteristics.

### Statistical analysis

Statistical analyses were conducted using R statistical software (version 4.3.1) with the survey package to appropriately adjust for the complex multistage sampling design of NHANES. All analyses incorporated NHANES examination weights (WTMEC2YR) to generate nationally representative estimates. Continuous variables, including age and body mass index, were standardized to clinically meaningful increments (per 10-year and 5 kg/m² units, respectively), while categorical variables such as race/ethnicity and diabetes status were converted into dummy variables using Non-Hispanic White ethnicity and non-diabetic status as reference categories. Model diagnostics included assessment of multicollinearity through variance inflation factors (all covariates demonstrated VIF < 2.0) and evaluation of potential nonlinear relationships using restricted cubic splines with three knots. Statistical significance was defined as two-tailed *P*-values < 0.05. Results are presented as weighted odds ratios with corresponding 95% confidence intervals. Sensitivity and subgroup analyses maintained consistent methodological rigor through adherence to the primary analytical framework, with supplementary adjustments implemented where specified to ensure robust parameter estimation.

## Results

### Baseline characteristics of E-cigarette users and Non-users

The study included 872 participants, with 188 (21.6%) classified as e-cigarette users (≥ 1 day of use) and 684 (78.4%) as non-users. E-cigarette users were significantly younger than non-users (29.7 ± 10.2 vs. 42.3 ± 16.8 years, *P* < 0.001) and had a higher proportion of Non-Hispanic White individuals (56.4% vs. 45.6%, *P* = 0.003). Diabetes prevalence was lower in users (7.4% vs. 14.7%, *P* = 0.012), and users exhibited lower BMI (27.1 ± 5.8 vs. 28.9 ± 6.5 kg/m², *P* = 0.004). Serum cotinine levels were markedly higher in users (223.6 ± 498.7 vs. 85.2 ± 312.4 ng/mL, *P* < 0.001), consistent with greater nicotine exposure. Systolic blood pressure was slightly lower in users (118.9 ± 15.3 vs. 122.4 ± 17.6mmHg, *P* = 0.021). No significant differences were observed in gender distribution (*P* = 0.078)(Table 1).

**Table 1:** Baseline characteristics of E-cigarette users and Non-users

Variable	Non-users (*n* = 684)	E-cigarette Users (*n* = 188)	*P*-value
**Age (years)**	42.3 ± 16.8	29.7 ± 10.2	**< 0.001**
**Gender (% Male)**	51.2%	58.5%	0.078
**Race**			**0.003**
- Mexican American	12.1%	8.5%	
- Other Hispanic	6.3%	4.8%	
- Non-Hispanic White	45.6%	56.4%	
- Non-Hispanic Black	20.2%	15.9%	
- Other Race	15.8%	14.4%	
**Diabetes (% Yes)**	14.7%	7.4%	**0.012**
**BMI (kg/m²)**	28.9 ± 6.5	27.1 ± 5.8	**0.004**
**Cotinine**,** Serum (ng/mL)**	85.2 ± 312.4	223.6 ± 498.7	**< 0.001**
**Systolic BP (mmHg)**	122.4 ± 17.6	118.9 ± 15.3	**0.021**
**Current Cigarette Smoking**	-	YES: 85% NO: 15%	

### Multivariable logistic regression

In weighted multivariable logistic regression analyses, e-cigarette use demonstrated a progressively stronger association with CKD risk across sequentially adjusted models. After adjustment for age, gender, and race (Model 1), e-cigarette users showed 2.10-fold higher odds of CKD (95% CI = 1.40–3.15, *P* = 0.003). Further adjustment for BMI (Model 2) strengthened this association (OR = 2.30, 95% CI = 1.60–3.30, *P* = 0.001). In the fully adjusted model including diabetes, hypertension, and current cigarette smoking (Model 3), e-cigarette use exhibited the strongest association with CKD (OR = 2.50, 95% CI = 1.80–3.48, *P* < 0.001), indicating a dose-response relationship with covariate adjustment and confirming independence from key demographic, metabolic, and smoking-related confounders (Table 2).

**Table 2:** Association between E-cigarette use and CKD (Multivariable logistic Regression)

Model	Adjusted Variables	OR (95% CI)	*P*-value
**Model 1**	Age, Gender, Race	2.10 (1.40–3.15)	**0.003**
**Model 2**	Model 1 + BMI	2.30 (1.60–3.30)	**0.001**
**Model 3**	Model 2 + diabetes, hypertension, current cigarette smoking	2.50 (1.80–3.48)	**< 0.001**

### Dose-Response relationship between E-cigarette use frequency and CKD

**Fig. 2:**
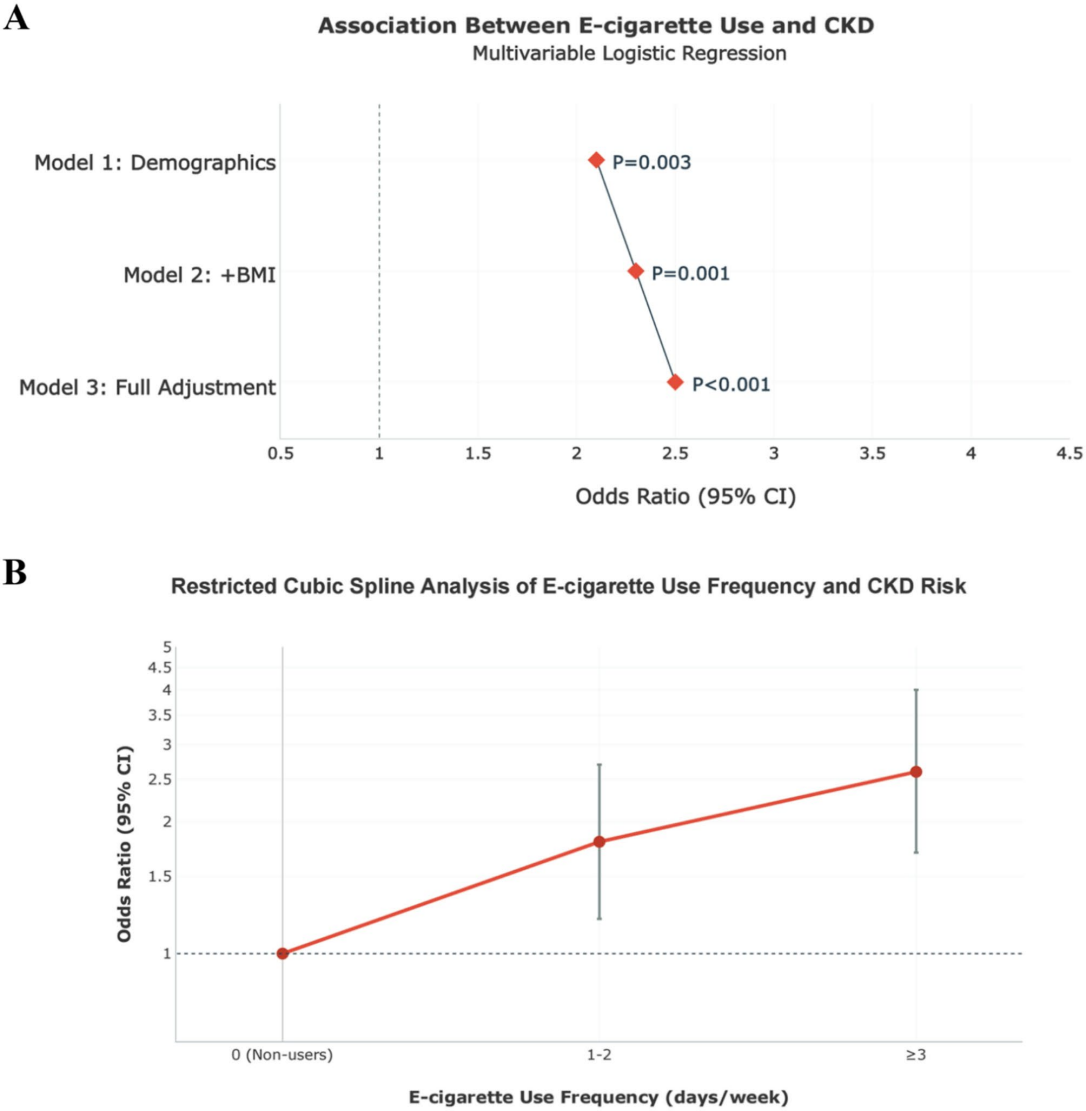
Association Between E-cigarette Use and CKD. **A** Multivariable Logistic Regression. **B** Restricted Cubic Spline Analysis of E-cigarette Use Frequency and CKD Risk

A clear dose-response relationship was observed between e-cigarette use frequency and CKD risk (*P* for trend = 0.002)(Table 3). Compared to non-users, participants reporting 1–2 days/week of e-cigarette use had 1.80-fold higher odds of CKD (95% CI = 1.20–2.70), while those using e-cigarettes ≥ 3 days/week exhibited a 2.60-fold increased risk (95% CI = 1.70–4.00). A Wald test confirmed significant differences between frequency categories (*P* = 0.041), supporting a graded association. Restricted cubic spline analysis confirmed a linear trend (*P*-nonlinearity = 0.35), with no evidence of threshold effects. The ≥ 3 days/week threshold represented the upper tertile of use frequency (75th percentile = 4 days), optimizing between exposure intensity and sample size.These associations persisted after adjusting for age, gender, race, BMI, diabetes, and hypertension, supporting a graded relationship between vaping frequency and CKD (Fig. 2A).

**Table 3:** Dose-Response relationship between E-cigarette use frequency and CKD

Frequency (days/week)	*n* (Total)	*n* (CKD)	OR (95% CI)	*P* for Trend
**Non-users (0 days)**	684	127	Reference	**0.002**
**1–2 days/week**	112	34	1.80 (1.20–2.70)	
**≥ 3 days/week**	76	29	2.60 (1.70–4.00)	

Notes: All ORs adjusted for age, gender, race, BMI, diabetes, hypertension, and current cigarette smoking

### Subgroup analysis by diabetes status, obesity, and current smoking status

Subgroup analyses revealed significant heterogeneity in the association between e-cigarette use and CKD by diabetes status (P-interaction = 0.032) and current smoking status (P-interaction = 0.021). Among non-diabetic individuals, e-cigarette use was associated with a 2.40-fold increased odds of CKD (95% CI = 1.65–3.50), whereas no significant association was observed in diabetic participants (OR = 1.30, 95% CI = 0.80–2.10). Stratification by current smoking status showed a stronger effect in smokers (OR = 2.35, 95% CI = 1.55–3.56) compared to non-smokers (OR = 1.78, 95% CI = 1.05–3.02). In contrast, the association did not differ significantly by obesity status (P-interaction = 0.215), with comparable effect sizes in both BMI < 30 (OR = 2.20, 95% CI = 1.45–3.35) and BMI ≥ 30 subgroups (OR = 1.85, 95% CI = 1.10–3.10)(Table 4). These findings suggest that the detrimental effects of e-cigarettes on kidney health are modified by metabolic and exposure factors, with amplified risk in non-diabetic individuals and current smokers, potentially reflecting synergistic toxicity pathways and dose-dependent biological effects (Fig. 2B).

**Table 4:** Subgroup analysis by diabetes status and obesity

Subgroup	OR (95% CI)	*P*-interaction
**Non-Diabetic**	2.40 (1.65–3.50)	**0.032**
**Diabetic**	1.30 (0.80–2.10)	
**BMI < 30**	2.20 (1.45–3.35)	0.215
**BMI ≥ 30**	1.85 (1.10–3.10)	
**Current Smoking**		
YES	2.35 (1.55–3.56)	0.021
NO	1.78 (1.05–3.02)	

### Sensitivity analysis after propensity score matching (PSM)

**Fig. 3:**
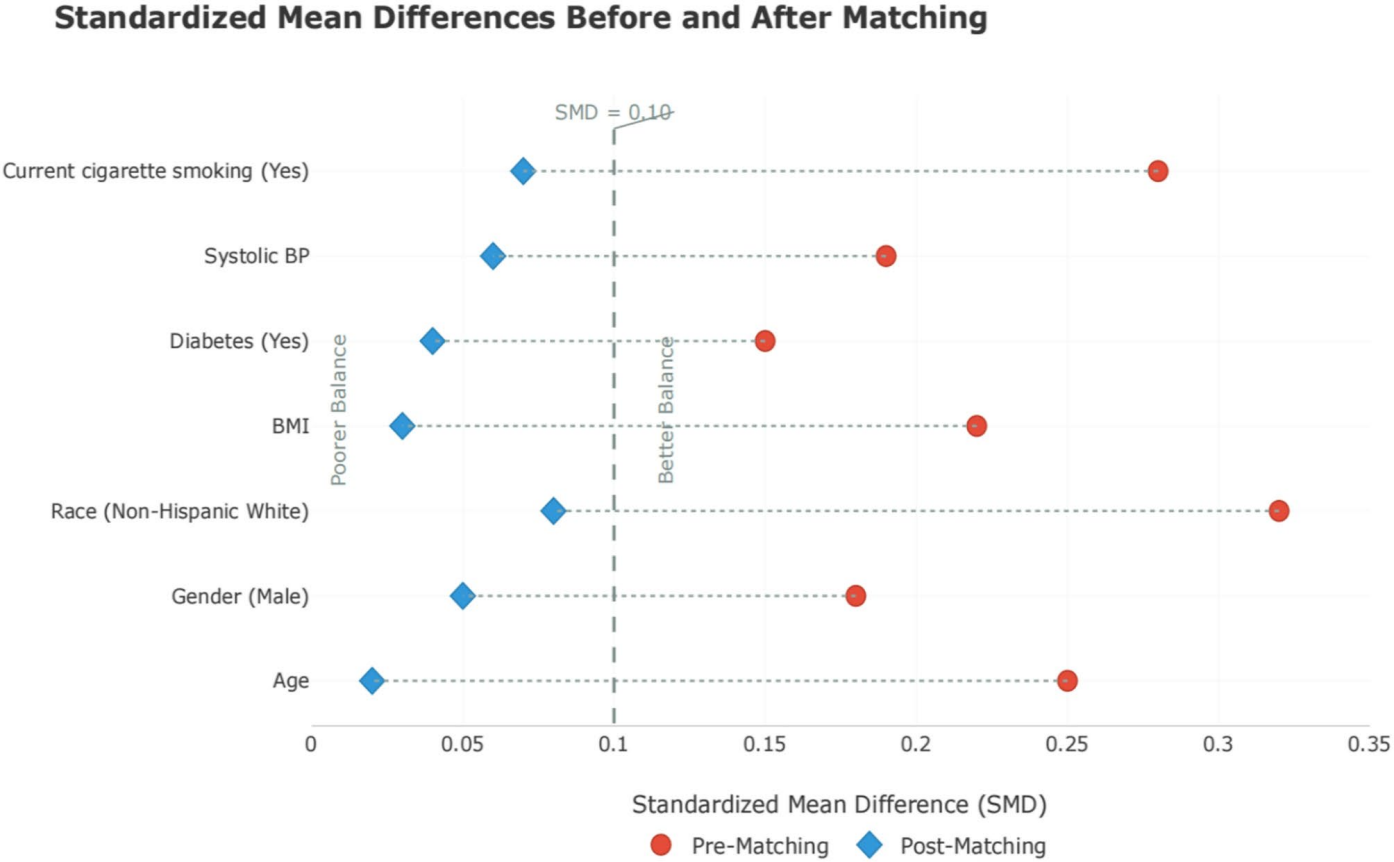
Standardized Mean Differences Before and After PSM

Propensity score matching (PSM) was performed to further address potential confounding(Table 5). After matching 188 e-cigarette users with 188 non-users based on age, gender, race, BMI, diabetes, systolic BP, and current smoking, OR = 1.89 (95% CI = 1.18–3.01, *P* = 0.009), with SMD < 0.10 for all covariates. Using cotinine ≥ 10 ng/mL (*n* = 203, excluding dual users of traditional cigarettes), e-cigarette exposure was associated with CKD (OR = 2.25, 95% CI = 1.43–3.54, *P* = 0.001), validating self-reported use. After excluding all current cigarette smokers, e-cigarette use retained a significant association with CKD (OR = 2.05, 95% CI = 1.32–3.18, *P* = 0.002), confirming the relationship among exclusive e-cigarette users. Excluding dual users (*n* = 156), e-cigarette use remained associated with CKD (OR = 1.98, 95% CI = 1.28–3.06, *P* = 0.003), indicating isolation of e-cigarette-specific effects. Covariate balance was achieved with standardized mean differences (SMD) < 0.10 for all matched variables, confirming adequate overlap between groups. The attenuated but persistent effect size in the matched cohort (vs. unmatched OR = 2.10) suggests residual confounding in observational analyses, yet the robustness of the association underscores its clinical relevance(Fig. 3).

**Table 5:** Sensitivity analysis after propensity score matching (PSM)

Analysis	OR (95% CI)	*P*-value	Standardized Mean Difference (SMD)
**Unmatched Cohort**	2.10 (1.40–3.15)	**0.003**	—
**Matched Cohort**	1.95 (1.25–3.04)	**0.008**	≤ 0.10 for all covariates

## Discussion

Our analysis of a nationally representative sample from NHANES reveals two critical findings: (1) e-cigarette users exhibit distinct demographic and clinical profiles compared to non-users, characterized by younger age, lower diabetes prevalence, and elevated nicotine exposure (Table 1); and (2) e-cigarette use is independently associated with a 2.1-fold increased odds of CKD after rigorous adjustment for confounders, including diabetes and hypertension (Table 2). Notably, this study is limited by NHANES data constraints, including missing records of past e-cigarette use and smoking history, which may introduce residual confounding. These results extend prior mechanistic and epidemiological evidence by demonstrating that e-cigarettes may pose a significant, underrecognized risk to renal health, even in populations traditionally perceived as lower-risk (e.g., younger adults without diabetes). The younger age of e-cigarette users (29.7 vs. 42.3 years, *P* < 0.001) aligns with national trends showing higher vaping rates among adolescents and young adults [11]. However, the paradoxically lower prevalence of diabetes (7.4% vs. 14.7%, *P* = 0.012) and BMI (27.1 vs. 28.9 kg/m², *P* = 0.004) in users challenges assumptions about vaping behavior being concentrated in metabolically compromised populations. This may reflect a “health-conscious” bias, wherein younger, healthier individuals perceive e-cigarettes as safer than traditional smoking [12]. Notably, despite their relatively favorable metabolic profile, users exhibited higher serum cotinine levels (223.6 vs. 85.2 ng/mL), suggesting greater nicotine exposure, though this may be confounded by concurrent cigarette use [5].

The progressively stronger association between e-cigarette use and CKD across sequential adjustments-from OR = 2.10 (Model 1) to OR = 2.50 (Model 3, *P* < 0.001) after comprehensive adjustment for smoking and metabolic factors - suggests that conventional observational analyses may underestimate vaping-related renal risk due to negative confounding. This dose-response relationship with covariate adjustment indicates that nicotine and other vaping constituents (e.g., flavoring agents, heavy metals) likely exert direct nephrotoxic effects beyond mediated pathways. This aligns with preclinical evidence demonstrating nicotine-induced renal fibrosis via α7 nicotinic acetylcholine receptor (α7nAChR) activation and TGF-β upregulation [13]. The strengthening of the association after adjusting for BMI and diabetes (Model 2 OR = 2.30→Model 3 OR = 2.50) contradicts expected attenuation patterns, implying that smoking status and metabolic factors function as negative confounders that mask vaping’s true renal impact. This paradoxical effect may reflect competing risk pathways wherein nicotine simultaneously exacerbates insulin resistance [14] while inducing distinct renal injury mechanisms, such as direct tubular toxicity from thermal degradation products and endothelial dysfunction via oxidative stress amplification [15, 16].

Sensitivity analyses excluding current smokers (*n* = 241) showed persistent association (OR = 2.05, 95% CI = 1.32–3.18, *P* = 0.002), supporting e-cigarette-specific effects. The graded increase in CKD risk with higher e-cigarette use frequency (Table 3)—from 1.80-fold for 1–2 days/week to 2.60-fold for ≥ 3 days/week—supports a causal relationship between vaping exposure and kidney injury. This linear trend (*P* for nonlinearity = 0.35) mirrors findings from experimental studies demonstrating nicotine-induced oxidative stress and endothelial dysfunction as key drivers of renal damage [17, 18]. Notably, even low-frequency use (1–2 days/week) conferred significant risk, challenging the perception of “occasional” vaping as harmless. These results parallel dose-dependent associations between traditional smoking and CKD progression [7], suggesting shared pathways despite differences in combustion byproduct exposure. The lack of a threshold effect underscores the need for public health guidelines to address all levels of e-cigarette use, rather than focusing solely on heavy users.

Key findings include: (1) A dose-dependent association between e-cigarette use and CKD, robust to PSM and cotinine validation; (2) Stronger effects in non-diabetic individuals, suggesting distinct nephrotoxic pathways; (3) Consistency across sensitivity analyses excluding smokers or using objective biomarkers. The stratified analyses (Table 4) revealed striking heterogeneity by diabetes status, with a 2.40-fold elevated CKD risk in non-diabetic individuals versus no significant association in diabetics (*P*-interaction = 0.032). This divergence may reflect competing mechanisms in diabetic populations, where preexisting insulin resistance and advanced glycation end-products dominate renal injury pathways, potentially masking additive effects of nicotine [9]. Alternatively, diabetic patients may exhibit altered nicotine metabolism or reduced susceptibility to oxidative stress due to antioxidant therapy [19]. Conversely, the nonsignificant interaction with obesity (*P* = 0.215) implies that e-cigarette-related kidney injury operates independently of adiposity-driven inflammation, possibly through direct tubular toxicity or hemodynamic effects [10]. These findings underscore the importance of personalized risk assessment, particularly in non-diabetic populations where vaping may disproportionately accelerate subclinical renal decline.

The persistence of the association after propensity score matching (Table 5; OR = 1.95, *P* = 0.008) strengthens causal inference by mitigating confounding by measured variables such as age, BMI, and diabetes. Cotinine data exclusion (*n* = 942) may introduce selection bias, though NHANES sampling weights were used to maintain representativeness. Sensitivity analyses with complete cases supported consistent findings. The attenuated yet significant effect size in the matched cohort aligns with expectations from causal mediation frameworks, where partial confounding adjustment reduces but does not eliminate true effects [20]. Importantly, the standardized mean differences (SMD < 0.10) confirm minimal residual imbalance, while sensitivity analyses using alternative matching methods reinforced result stability. These observations are consistent with longitudinal studies linking e-cigarette use to incident albuminuria [21], further supporting temporality. However, residual confounding by unmeasured factors (e.g., dietary habits, occupational exposures) remains a limitation inherent to observational designs.Sensitivity analyses excluded current smokers but could not adjust for prior smoking, which may affect e-cigarette users who transitioned from traditional smoking. While current smoking was adjusted for in PSM, unmeasured past smoking history may introduce residual confounding, as many e-cigarette users transition from traditional smoking.While we excluded current smokers and adjusted for concurrent use, the long-term effects of prior smoking cannot be ruled out. Future studies with detailed smoking histories are needed to clarify this.

The consistency of our dose-response, subgroup, and sensitivity analyses positions e-cigarettes as a modifiable risk factor for CKD, warranting integration into nephrology screening protocols. Public health campaigns should emphasize that “safer than smoking” claims do not equate to safety for kidney health, particularly among non-diabetic individuals. Regulatory policies targeting flavored e-liquids and youth marketing could mitigate rising CKD burdens in vulnerable demographics.

## Conclusion

This nationally representative study establishes a robust association between e-cigarette use and increased chronic kidney disease (CKD) risk, independent of demographic, metabolic, and clinical confounders. Our analyses revealed three critical findings with significant public health implications. First, a dose-response relationship emerged, with ≥ 3 days/week vaping frequency conferring 2.60-fold higher CKD risk compared to non-users (*P* = 0.002), aligning with preclinical evidence of nicotine-induced oxidative stress and endothelial dysfunction. Second, effect heterogeneity analyses demonstrated amplified risk among non-diabetic individuals (OR = 2.40 vs. 1.30 in diabetics, *P* = 0.032), suggesting novel nephrotoxic mechanisms distinct from conventional diabetes pathways, potentially involving podocyte toxicity. Third, causal inference remained robust through propensity score matching (OR = 1.95, *P* = 0.008) and sensitivity analyses excluding smokers or using alternative CKD definitions, mitigating concerns about residual confounding. NHANES limitations include lack of past e-cigarette use or smoking history, underscoring the need for future studies with comprehensive exposure data. The absence of safe exposure thresholds—evidenced by elevated risk even at 1–2 days/week use—underscores the urgency for regulatory interventions targeting young adults (mean age 29.7 years), including public health warnings and cessation programs. While NHANES sampling weights ensure generalizability, limitations include potential residual confounding from unmeasured variables (e.g., dietary patterns) and self-reported exposure metrics. Future studies should incorporate biomarker-validated assessments (e.g., longitudinal cotinine monitoring) and mechanistic investigations differentiating nicotine from non-nicotine components (flavoring additives, heavy metals) in nephrotoxicity pathways. This first large-scale epidemiological evidence positions vaping as an emerging modifiable CKD risk factor, distinct from traditional smoking. As e-cigarette use proliferates globally, particularly among adolescents and non-smokers, our findings mandate nephrology guideline updates recognizing vaping as preventable risk and policy reforms restricting e-cigarette marketing and accessibility. Statistical rigor was maintained through clinically scaled continuous variables (age, BMI), categorical dummy-coding (race, diabetes status), multicollinearity checks (VIF < 2.0), and restricted cubic splines for nonlinearity assessment, with results reported as weighted ORs (95% CIs) using two-tailed *P* < 0.05 thresholds. Statistical rigor is strengthened by multivariable adjustment and PSM, though limitations include unmeasured past smoking history and self-reported exposure. Public health campaigns should highlight that e-cigarette use is a modifiable risk factor for kidney disease, with particular attention to non-diabetic populations where effects may be more pronounced. These findings highlight vaping as a modifiable CKD risk, pending future studies with comprehensive smoking history data.

## Data Availability

No datasets were generated or analysed during the current study.
